# Validation of a Self-Perceived Adaptive Behaviors Scale in Older Chilean Women and Percentiles for Evaluation

**DOI:** 10.3390/ijerph18020731

**Published:** 2021-01-16

**Authors:** Rossana Gomez-Campos, Ruben Vidal-Espinoza, Luis Felipe Castelli Correia de Campos, Cynthia Lee-Andruske, Jose Sulla-Torres, Carolina Cornejo-Valderrama, Nancy Lepe-Martinez, Juan Lagos-Luciano, Rodrigo Monne de la Peña, Camilo Urra-Albornoz, Paz Pezoa-Fuentes, Marco Cossio-Bolaños

**Affiliations:** 1 Universidad Católica del Maule, Avenida San Miguel 3605, 3466706 Talca, Chile; ccornejo@ucm.cl (C.C.-V.); nlepe@ucm.cl (N.L.-M.); jlagos@ucm.cl (J.L.-L.); rmonne@ucm.cl (R.M.d.l.P.); ppezoa@ucm.cl (P.P.-F.); 2 Universidad Católica Silva Henriquez, Gral. Jofré 462, 8330225 Santiago, Chile; rvidale@gmail.com; 3 Universidad del Bio Bio, Avda. Collao 1202 Casilla 5-C, 4050231 Chillán, Chile; lcastelli@ubiobio.cl; 4 Centro de Investigación CINEMAROS, Calle San Domingo 311, 04000 Arequipa, Peru; candruske@gmail.com; 5 Universidad Nacional San Agustín de Arequipa, Av. Venezuela s/n, 04000 Arequipa, Peru; jsulla@unsa.edu.pe; 6 Escuela de Kinesiología, Facultad de Salud, Universidad Santo Tomás, Carlos Schorr 255, 3460000 Talca, Chile; camilo.urra.albornoz@gmail.com

**Keywords:** adaptive behavior, older adults, women, body adiposity, physical fitness

## Abstract

Healthy ageing means optimizing opportunities that allow older individuals to participate actively in society without discrimination. Learning adaptive behaviors (AB) may be extremely important for individuals for all stages of life. The goal of this study was: (a) to create a scale for self-perceived adaptive behavior, and (b) propose percentiles for evaluating AB in older adult women. A self-perception scale was created to measure adaptive behavior. Anthropometric and physical fitness variables for 192 older Chilean women (ages 60 to 88) were collected and evaluated. Content validity reflected agreement from 0.75 to 1.0. Construct validity carried out with exploratory factor analysis (EFA) resulted in 11 dimensions with 62 items in groups. Saturation oscillated between 0.62 and 0.85 with the explanation of variation as 46.27%. Cronbach’s Alpha was r = 0.83. The results indicated that the scale developed was valid and reliable for the Chilean women studied. This scale may be used to measure self-perception of AB patterns in older women. Furthermore, the percentiles allow for classification of the AB by age and anthropometric indices.

## 1. Introduction

Access to adaptive behavior (AB) refers to the person’s ability to assume more and more responsibility for his or herself and to help others in developing skills for daily living [1]. Additionally, it has been established as the effectiveness and the degree to which the individual meets the standards of personal independence and social responsibility [2] (p. 11).

Learning AB and the accompanying skills may be some of the most important life skills, not only during childhood [1], but also during other stages of life. Without the continual help of these adaptive behaviors, individuals are left with adaptive deficits that limit them in functioning in one or more activities in daily life, such as communication, social interaction, and independent living in multiple environments, including the home, school, work, and the community [3].

In fact, successful ageing requires effective adaptation that at the same time implies the flexible use of strategies to optimize personal functioning and well-being during this life stage [4]. This involves the ability to perform activities of daily living. These allow individuals to care for their health and personal safety, dress and bathe themselves, communicate, behave in a socially acceptable way, use academic, recreational, and work skills effectively, and participate in a community lifestyle [5]. These skills are required by individuals in order to meet their personal needs and confront social and natural demans they encounter in social environments [6].

In this context, the novelty of using AB with older adults is that it could be used to evaluate, diagnose, and track the progress of the changes in behavior during the aging process [7,8]. Therefore, it is important to evaluate those daily activities that are culturally sensitive and age appropriate [9]. This information could help older adults to improve skills where they have greater weaknesses while improving their AB.

Recently, it has been highlighted that AB scales are important for diagnosing, intervening, and planning [6], independent from age and sex. In this sense, an urgent need exists to identify and develop easy to use instruments, especially for incorporating into daily routines of health care professionals [10], following up on functional changes in individuals [11], understanding the person, and providing appropriate life experiences [12], among other properties.

Consequently, an AB instrument, specifically for older adult women, may help identify the presence of deviations from the norms as well as identify areas for evaluation and intervention for those most affected [13]. This is relevant for the population undergoing the ageing process. As individuals age, through the experiences throughout the life span, they acquire ways of dealing with the environment, economic and social resources, relationships, and assistance systems. These may profoundly affect well-being due to longevity [14]. During this stage, the ageing process involves the deterioration of the musculoskeletal system, decreased glomerular filtration, low pulmonary ventilation, glucose intolerance associated with age, loss of hearing, vision, memory, and motor coordination abilities as well as degenerative diseases [15].

Various studies have shown that the adaptation process is associated positively with the indicators of successful ageing. These indicators include: well-being, life satisfaction, daily living skills, communication, socialization, and quality of life [16,17,18]. However the instruments proposed from some of these studies do not include a number of dimensions, such as leisure, recreational spaces, self-direction, and physical fitness. These dimensions need to be included in the same instrument in order to assess the AB of older adult women.

During the past decade, Latin America has undergone a demographic transition as well as a rapid epidemiological transition [19]. Chile also finds itself in a full scale demographic transition process where the adult population is ageing rapidly [20]. Moreover, in recent years, health and government professionals have warned that psychological health, social relationships, and economic conditions have become primary determinants for the deterioration of the quality of life for older adults [21].

This information highlights the necessity to develop an instrument for measuring AB of older adults in South America and Chile. The psychometric properties of this instrument need to include social and cultural characteristics specific to the ageing adult population. For example, for older adults, the following dimensions need to be taken into consideration: communication, safety, leisure, use of recreational spaces, self-care, self-direction, home life, functional skills, socialization, health, and functioning capacity. These need to be constantly evaluated. In addition, activities of daily living require different constructs that all humans need to live successfully. These dimensions include basic skills for fulfilling personal needs as well as for confronting social and natural demands in the surrounding environment [6].

In fact, without adaptive behavior skills, older adults, especially older women, have limited independent functioning and increased difficulties in their daily living activities [4]. Therefore, evaluating AB based on the aforementioned dimensions could contribute to providing optimum opportunities for older adults to participate actively in society without experiencing discrimination while enjoying an independent and fulfilling quality of life [22].

Thus, the principal objective of this study was to (a) create a scale for self-perceived AB for older adult Chilean women. The secondary objectives were to (b) propose percentiles for evaluating a diagnosing AB.

## 2. Methods

### 2.1. Type of Study and Sample

A cross sectional descriptive research project was designed to study 192 older adult Chilean women between the ages of 60 and 88 years old. The age ranges were based on those used by the Municipality of Talca (Chile), enrolling self-supporting adult women between the ages of 60 to 90 years old. The sample was selected by convenience non-probabilistically. For this type of study where accurate estimations are required for the coefficients of exploratory factor analysis (EFA), in general, the sample size is sufficient with 150 to 200 cases [23].

Anthropometric and physical fitness variables were evaluated. Additionally, the women were given a questionnaire of 74 questions to answer about self-perception adaptive behaviors. All of the subjects belonged to six clubs for older adults from the Region of Talca (Chile). The women were informed about the objectives of the study and the variables to be evaluated.

Women signing the informed consent form, completing all of the tests, and performing daily activities independently were included in the study (shopping, walking, knitting, dancing, paying bills, among others). Those excluded from the research were women not in the established age range and those with any type of injury that might prevent them from completing the physical tests. The Ethics Committee of the Universidad Católica del Maule (Catholic University of Maule) (Chile) approved the project (UCM 105-2018).

### 2.2. Procedures

Each woman provided her own birth date information (day, month, year), and this was calculated to the decimal subtracting the evaluation date. Data collection was carried out between April and July 2018 in the laboratory at the Catholic University of Maule (Chile). Initially, the Self-perception Adaptive Behavior Scale for Older Women (ABSOW-I [EACAM-I: escala de conducta adaptativa para mujeres de tercera edad]) was evaluated, followed by collection of anthropometric variables, and results from the physical fitness tests. The entire process was carried out by 4 evaluators previously trained in measurement techniques and surveys [24,25].

The survey was used to measure the AB values. A scale was created with 11 dimensions (communication, use of community resources, home life, health, safety, self-care, functional skills, leisure, self-direction, socialization, and functional ability). These dimensions are relatively similar to the scales proposed for children and adolescents with mental disabilities [1,12]. However, we included less questions and fewer alternatives for each question. Initially, the scale consisted of 74 questions. To further develop the instrument, theoretical research information was collected from various databases (PubMed, Scopus). Then, the resulting literature was systematically analyzed in order to support the 11 dimensions and the corresponding questions. The instrument had three possible choices. These varied according to the directionality of the question, for example, [(a) always, (b) sometimes, (c) never]; [(a) enough, (b) little, (c) nothing]; and [(a) high, (b) average, and (c) low]. Each questionnaire was given to each woman to answer individually, and it took each person approximately 25–30 min to complete.

The anthropometric variables collected included: weight, height, and neck circumference. The variables were evaluated according to the recommendations of the International Society for the Advancement of International Kinanthropometry [26]. Body weight (kg) was measured with a digital scale (SECA, BMI 804, Hamburg, Germany) with an assessed of 0.1 kg. Standing height was measured with a stadiometer (SECA, 203) with an accuracy of 0.1 cm. Neck circumference (NC) and waist circumference (WC) were measured (cm) with a nylon tape measure (SECA) with an accuracy of 1.0 mm. Body Mass Index (BMI) was calculated by using the formula: [BMI = weight (kg)/height (m)^2^]. The anthropometric measurements were taken twice the same day. The technical error of measurement (TEM) was less than 1.3%.

### 2.3. Validity and Reliability of the Scale

Validity was determined using two methods (content validity and construct validity). Content validity was verified by using expert judges in keeping with Wierseman’s [27] suggestions. A call was put out for eight professionals in the content areas. However, only five responded and performed the role of expert judges for the scale (02 from psychology, 01 physical education, and 02 from gerontology). In addition to their expertise in their disciplines, these five experts also met the selection criteria in terms of academic training, content experience, and community recognition in their different fields. A separate file was created for each expert where the professional analyzed the degree of representation, relevance, diversity, clarity, simplicity, and comprehensiveness for each of the items for the instrument created. Using a scale of 1 to 5 points, individually, the experts assigned a value to each of the items of the instrument (equivalent to the values of not important to very important). Based on Aiken’s V coefficient and its confidence intervals [28], the degree the items reflected the content areas was indicted as an adequate proportion of the construct [29], Items with values greater than V of Aiken ≥0.75 were included [30].

Validation of the instrument by its construct was carried out with the technique of factor analysis using EFA to determine the underlying structure of the data [31]. Prior to carrying out the factor analysis, Kaiser Meyer-Olkin’s (KMO) criteria of sample adequacy and Bartlett’s sphericity value were considered to establish the relevance of the factor analysis. The factor structure of the scale was evaluated using the option of the principal components method and the varimax rotation to maximize the independence between the factors. Factor analysis provided the explained variance measure and the eigenvalues for each item. To verify the instrument’s reliability, internal consistency (Cronbach’s Coefficient) was analyzed by using Spearman-Brown and Guttman’s formula [32].

### 2.4. Statistics

The normal distribution of the data was tested using the Kolmogorov-Smirnov test. We tested several transformations to achieve data normality and homogeneity. However, no approaches produced effective outcomes. Therefore, a non-parametric multiple comparison test and Bonferroni’s correction were performed when necessary. The results were expressed as mean, standard deviation (mean ± SD), and range (minimum and maximum values).

Content validity was determined by calculating the averages of each item and the V of Aiken test [33] based on the quantitative values the expert judges assigned to each of the items. Construct validity was obtained through Exploratory Factor Analysis (EFA). As described previously, to determine if factor analysis was effective, Kaiser Meyer-Olkin’s test for sample adequacy and Bartlett’s sphericity test were performed. Afterwards, exploratory factor analysis (EFA) was carried out with the principal components method and varimax rotation. Reliability was calculated with Cronbach’s alpha. In all cases, the level of significance adopted was *p* < 0.05. The smoothed distribution of percentiles was constructed using the LMS method [34]. The percentiles p5, p10, p15, p25, p50, p75, p85, and p90 were calculated by age range, BMI range, and NC (neck circumference). The maximum penalty probability procedure was used to create three smoothed curves: (t) Box-Cox Power, M(t) median, and S(t) Coefficient of variation. The calculations were carried out with LMS Chartmaker Pro version 2.3 software program (The Institute of Child Health, London). For all tests, statistical significance was established at *p* < 0.05. The data for this study were processed using SPSS Statistics for Windows, version 18.0 (SPSS Inc., Chicago, IL, USA).

## 3. Results

The anthropometric variables and AB dimensions of older adult women are illustrated in Table 1. The age range of the women studied varied from 60.2 to 88.9 years of age. ABSOW-I (EACAM-I) shows the 11 dimensions.

Values for the V of Aiken test are presented in Table 2. The values for each question varied from 0.75 to 1.0 while the values for the categories were between 0.77 and 0.94. For all of the cases, the values obtained from the judges reflected an agreement of 0.75 to 1.0.

The construct validity values (exploratory factor analysis, EFA) and reliability are illustrated in Table 3. The corresponding value for Kaiser Meyer-Olkin’s (KMO) test was 0.653. Bartlett’sspherecity test resulted in a significance of (x^2^ = 3517.477; *p* < 0.001). According to Siembida et al. [35], after performing the extraction, the communalities must be greater than 0.30 to assume that the measurement has a good validity. Factor analysis provided the measure of % of explanation of the variance. The result was 46.27%. Proper Values (PV) or Eigen values were greater than 1.62 in the 11 dimensions. Saturation values varied from 0.62 to 0.85 for the 62 questions. A factor weight greater than or equal to 0.40 was considered as an inclusion criterion [36], leaving the items grouped into 11 dimensions. Eliminating the items was also supported by the results from the analysis of the reliability analysis. For each question, Cronbach’s Alpha showed values from 0.70 to 0.88, and for the total instrument, the alpha was r = 0.83.

The percentiles of the ABSOW-I (EACAM-I) by age, BMI and NC, are itemized in Table 4. As indicated in the table, the percentiles in the scale decreased gradually as age, BMI, and NC.

## 4. Discussion

The results from this research have shown that the instrument created demonstrated both content and construct validity (EFA). Based on the expert judges’ analysis of the content, the Aiken values obtained in this study were valid. These findings reflected homogeneity among the five judges. As suggested by Wierseman [27], the homogeneity allowed for proving relevance, diversity, clarity, simplicity, and comprehensiveness of the instrument.

In fact, after excluding the 14 questions with values less than 0.74, 62 questions with values greater than 0.75 remained. The findings obtained from this study were like those of other research projects with similar characteristics [37,38,39]. These findings ensure an adequate measurement of ABSOW-I (EACAM-I) psychometric properties. However, despite being a necessary condition in terms of quality control, it is not sufficient for interpreting the test scores [40]. Therefore, these findings need to be contrasted with other techniques.

In this sense, this study used EFA where the results of the KM, first, indicated, if convenient, use of factor analysis for the proposed scale. In addition, the index values for Bartlett’s spherecity illustrated that factor analysis was valid for the scale. With regard to the saturation results obtained, they were greater than 0.62, based on/according to the recommendation to discard reagents with a low correlation [41]. The Varimax rotation method maximized the sum of the variance factor loads, minimizing the number of the original variables with high saturation for the factors extracted [42].

The findings reported in this current study are similar to scales and inventories that have been submitted for validation using EFA and for reliability using internal consistency [43,44,45]. The similar proof and values obtained allowed for confirmation of each of the items and, consequently, acceptance of the dimensions.

With regard to the percentiles that were developed, the LMS method was used for this study to generate smoothed curves for the age range, adiposity indicators (BMI and NC). In general, this type of tool serves to classify and/or diagnose an individual’s health status or that of a population [46]. In fact, the cut off points adopted for this study (<p15, as low; p15 to p85. as moderate; and >p85, as high). These cut off points were based on previous studies where questionnaires and/or scales were used [24,25]. This information may be used to classify and monitor the AB of older women. This information is relevant for health professionals since it may be used to identify older adults with low AB, to those who may suggest interventions based on age and/or body fat (BMI and NC).

The instrument developed in this study is relevant not only for contexts for working with older adults, but it is also useful for identifying AB based on age and dimensions. Therefore, an effective AB will allow an individual to fulfill expected personal and social responsibilities for his or her age and cultural group [47]. As well, it could be used as an indicator for adiposity as proposed in this study. Based on these points, we recommend using the instrument created in this research to identify AB to help improve communication patterns, use of community resources, home life, health, safety, self-care, functional skills, leisure, self-direction, socialization, and functional capacity, especially for older adult Chilean women as well as other age groups. The final instrument is attached in Spanish and English at the end of the manuscript (Supplementary Material). If researchers or other professionals are interested, calculations may be made quickly and accurately by using the following link: http://reidebihu.net/physicalolder.php.

This study has some limitations that need to be acknowledged. It was not possible to include in this research older men because they tend not to participate in the clubs for older adults. Therefore, only women were included in this research. Furthermore, sample selection was non-probabilistic, limiting generalizability of the results to other contexts. However, this study also made an important contribution. It is the first research study carried out on a national scale in Chile where an ABSOW-I (EACAM-I) has been proposed with 11 dimensions. Moreover, may be analyzed by age and body adiposity. The scale shows some adequate psychometric properties. The instrument is easy to administer; requires little time to fill out; and does not need qualified trained people to administer it. These characteristics make this scale an effective instrument to evaluate the self-perception to evaluate the AB in epidemiological studies. The scale with its 62 items demonstrated a high reliability and a high correlation between the items that guarantees that the internal structure of the instrument is solid [48]. This allows it to be administered to other population samples. However, despite showing an adequate factor result, reliability, and correlation between the items, this study does not exclude the existence of other results from different samples. Therefore, a better fit of the model needs to be evaluated in the future through confirmatory factor analysis.

## 5. Conclusions

The scale developed to assess the adaptive behavior of adult older women is valid and reliable. Furthermore, it can be used to measure self-perception AB patterns of independent older adult Chilean women. In addition, the percentiles allow classification of the AB by age and anthropometric indices. These may also be used routinely for older women.

## Figures and Tables

**Table 1:** Characteristics of the simple studied.

Variables	Mean	SD	Mínimum	Maximum
Age (years)	70.8	5.9	60.2	88.8
Anthropometry				
Weight (kg)	67.9	10.6	40.0	98.4
Height (cm)	152.3	5.5	135.0	170.0
Adiposity Indicators				
C. Neck (cm)	35.5	3.9	27.8	74.0
C. Waist (cm)	91.9	10.7	59.0	120.0
BMI (kg/m^2^)	29.1	4.4	14.9	43.7
**ABSOW-I (EACAM-I)**	**Median**	**Range**	**Mínimum**	**Maximum**
D. Communication	14.0	9.0	6.0	15.0
D. Resource use	15.0	11.0	8.0	19.0
D. Home life	15.0	10.0	5.0	15.0
D. Health	14.0	11.0	8.0	19.0
D. Safety	16.0	35.0	10.0	45.0
D. Self-care	16.0	8.0	10.0	18.0
D. Functional Skills	17.0	12.0	9.0	21.0
D. Leisure	14.0	9.0	9.0	18.0
D. Self-direction	19.0	12.0	9.0	21.0
D. Socialization	17.0	9.0	10.0	19.0
D. Functional Capactiy	15.0	8.0	10.0	18.0
Total ABSOW-I (EACAM-I)	169.0	76.0	134.0	210.0

Legend: SD: Standard deviation, D: Dimension, C: Circumference, ABSOW-I: Adaptive Behavior Scale for Older Women (EACAM-I: escala de conducta adaptativa para mujeres de la tercera edad).

**Table 2:** Content validity of the instrument created (ABSOW-I [EACAM-I]) using Aiken by question and dimension.

°	RP	RE	DI	CL	SI	CO	T	N°	RP	RE	DI	CL	SI	CO	T	N°	RP	RE	DI	CL	SI	CO	T
Comunication								Safety								Leisure							
1	1.00	0.75	0.86	0.86	0.86	1.00	0.89	22	0.93	0.96	0.75	0.86	1.00	1.00	0.92	41	1.00	0.86	0.75	0.86	0.86	0.86	0.87
2	0.93	0.93	0.75	0.93	0.93	0.93	0.90	23	1.00	0.86	0.75	0.86	0.93	0.93	0.89	42	0.93	0.93	0.96	0.75	0.75	0.75	0.85
3	0.79	0.96	0.96	0.86	0.82	0.86	0.88	24	1.00	0.86	0.75	0.86	0.86	0.86	0.87	43	0.86	0.82	0.86	0.96	0.96	0.86	0.89
4	0.86	0.82	0.75	0.93	1.00	0.75	0.85	25	0.93	0.93	0.96	0.75	0.75	0.75	0.85	44	0.75	0.86	0.75	0.86	0.75	0.75	0.79
5	0.93	0.71	0.79	0.82	0.93	0.96	0.86	26	0.86	0.82	0.86	0.96	0.96	0.96	0.90	45	0.86	0.82	0.86	0.96	0.96	0.96	0.90
T	0.90	0.83	0.82	0.88	0.91	0.90	0.87	T	0.94	0.89	0.81	0.86	0.90	0.90	0.88	46	0.86	0.82	0.86	0.96	0.96	0.86	0.89
																T	0.88	0.85	0.84	0.89	0.87	0.84	0.86
Use of recreational spaces								Self-care								Self-direction							
6	0.82	0.93	0.79	0.86	0.75	0.86	0.84	27	0.93	0.93	0.96	0.75	0.75	0.75	0.85	47	0.75	0.86	0.75	0.86	0.86	0.75	0.81
7	0.89	0.85	0.82	0.88	0.88	0.89	0.87	28	1.00	0.86	0.75	0.86	0.96	0.96	0.90	48	0.86	0.75	0.86	0.93	0.86	0.96	0.87
8	0.86	0.82	1.00	0.96	0.75	0.82	0.87	29	0.93	0.93	0.96	0.75	0.96	0.96	0.92	49	0.93	0.96	0.75	0.86	0.75	0.86	0.85
9	0.93	1.00	0.93	0.86	0.96	1.00	0.95	30	0.86	0.82	.86	0.96	0.86	0.82	0.86	50	1.00	0.86	0.75	0.86	0.96	0.93	0.89
10	0.82	0.93	0.86	0.93	0.86	0.93	0.89	31	0.75	0.86	0.75	0.86	0.75	1.00	0.83	51	0.93	0.96	0.75	0.86	0.86	0.82	0.86
11	0.89	0.85	0.82	0.88	0.88	0.90	0.87	32	0.86	0.75	0.86	0.93	0.96	0.93	0.88	52	0.93	0.96	0.75	0.86	0.86	0.82	0.86
T	0.87	0.90	0.87	0.90	0.85	0.90	0.88	33	0.93	0.96	0.75	0.86	0.86	0.90	0.88	T	0.90	0.89	0.77	0.87	0.86	0.86	0.86
								T	0.89	0.87	0.84	0.85	0.87	0.90	0.87								
Home life								Functional skills								Socialization							
12	1.00	0.75	0.75	0.86	0.75	0.75	0.81	34	0.75	0.86	0.75	0.86	0.75	1.00	0.83	53	1.00	0.86	0.75	0.86	0.86	0.86	0,87
13	0.93	0.96	0.96	0.75	0.82	0.96	0.90	35	0.86	0.75	0.86	0.93	0.86	0.90	0.86	54	1.00	0.86	0.75	0.86	0.86	0.86	0.87
14	0.86	0.86	0.86	0.96	1.00	0.86	0.90	36	0.93	0.96	0.75	0.86	0.75	0.90	0.86	55	0.93	0.93	0.96	0.75	0.75	0.93	0.88
15	0.75	0.75	0.75	0,86	0.93	0.93	0.83	37	0.86	0.75	0.86	0.93	0.96	1.00	0.89	56	0.86	0.82	0.86	0.96	0.96	0.82	0.88
16	1.00	0.75	0.75	0.86	0.90	0.86	0.85	38	0.93	0.96	0.75	0.86	0.86	0.82	0.86	57	0.75	0.86	0.75	0.86	0.96	1.00	0.86
T	0.91	0.81	0.81	0.86	0.88	0.87	0.86	39	0.86	0.75	0.86	0.93	0.75	1.00	0.86	T	0.91	0.87	0.81	0.86	0.88	0.89	0.87
								40	0.93	0.96	0.75	0.86	0.96	0.93	0.90								
								T	0.87	0.86	0.80	0.89	0.84	0.94	0.87								
Health																Functional capacity							
17	0.75	0.75	0.86	0.93	0.75	0.75	0.80									58	0.86	0.75	0.86	0.93	0.86	0.86	0,85
18	0.96	0.96	0.75	0.86	0.96	0.96	0.91									59	0.93	0.96	0.75	0.86	0.86	0.86	0,87
19	0.86	0.96	0.96	0.86	0.86	0.86	0.89									60	1.00	0.86	0.75	0.86	0.93	0.75	0,86
20	0.75	0.86	0.86	0.86	0.93	0.93	0.87									61	0.93	0.93	0.96	0.75	0.82	0.96	0,89
21	0.75	0.75	0.75	0.86	0.93	0.75	0.80									62	0.86	0.82	0.86	0.96	1.00	0.86	0,89
T	0.81	0.86	0.84	0.87	0.89	0.85	0.85									T	0.92	0.86	0.84	0.87	0.89	0.86	0.87

Legend: RP: Representativeness, RE: Relevance, DI: Diversity, CL: Clarity, SI: Simplicity, CO: Comprehensibility, T: Total.

**Table 3:** Exploratory factorial analysis values and reliability of the ABSOW-I (EACAM-I) for older women.

Dimensions/Factor	S	C	R	Dimensions/Factor	S	C	R	Dimensions/Factor	S	C	R	Dimensions/Factor	S	C	R
Comunication				Health				Functional skills				Socialization			
1	0.66	0.511	0.829	17	0.68	0.383	0.828	34	0.68	0.398	0.83	53	0.68	0.524	0.83
2	0.74	0.61	0.829	18	0.76	0.472	0.832	35	0.63	0.419	0.827	54	0.68	0.512	0.828
3	0.71	0.572	0.829	19	0.85	0.405	0.831	36	0.74	0.587	0.824	55	0.73	0.543	0.828
4	0.62	0.495	0.828	20	0.71	0.389	0.829	37	0.72	0.557	0.828	56	0.66	0.398	0.829
5	0.68	0.457	0.826	21	0.81	0.459	0.831	38	0.73	0.542	0.825	57	0.66	0.412	0.828
PV	6.8			PV	2.26			39	0.73	0.494	0.822	PV	1.62		
%V	10.98			%V	3.64			40	0.71	0.52	0.828	%V	2.51		
% cumulative	10.98			% cumulative	24.66			PV	1.9			% cumulative	43.57		
Reliability total			0.710	Reliability total			0.724	%V	3.5			Reliability total			0.717
								% cumulative	35.56						
								Reliability total			0.744				
Use of recreational spaces				Safety				Leisure				Functional capacity			
6	0.73	0.597	0.829	22	0.72	0.52	0.83	41	0.75	0.424	0.828	58	0.75	0.52	0.825
7	0.69	0.51	0.827	23	0.6	0.419	0.827	42	0.72	0.417	0.825	59	0.77	0.47	0.827
8	0.69	0.498	0.826	24	0.74	0.47	0.833	43	0.73	0.377	0.825	60	0.72	0.434	0.827
9	0.79	0.578	0.827	25	0.71	0.343	0.831	44	0.7	0.389	0.825	61	0.76	0.387	0.83
10	0.74	0.472	0.829	26	0.72	0.356	0.855	45	0.69	0.459	0.829	62	0.75	0.472	0.831
11	0.77	0.424	0.829	PV	2.23			46	0.66	0.389	0.828	PV	1.56		
PV	3.67			%V	3.6			PV	1.82			%V	2.70		
%V	5.93			% cumulative	28.26			%V	2.9			% cumulative	46.27		
% cumulative	16.91			Reliability total			0.712	% cumulative	38.46			Reliability total			0.717
Reliability total			0.763					Reliability total			0.806				
Home life				Self-care				Self-direction							
12	0.75	0.53	0.829	27	0.74	0.48	0.832	47	0.74	0.419	0.828				
13	0.78	0.42	0.829	28	0.7	0.382	0.829	48	0.79	0.459	0.83				
14	0.76	0.57	0.83	29	0.65	0.48	0.824	49	0.72	0.567	0.829				
15	0.79	0.41	0.829	30	0.73	0.383	0.829	50	0.73	0.498	0.827				
16	0.82	0.59	0.829	31	0.77	0.47	0.83	51	0.69	0.356	0.827				
PV	2,55			32	0.69	0.529	0.826	52	0.72	0.512	0.828				
%V	4,11			33	0.73	0.602	0.829	PV	1.79						
% cumulative	21.02			PV	2.05			%V	2.60						
Reliability total			0.726	%V	3.8			% cumulative	41.06						
				% cumulative	32.06			Reliability total			0.742				
				Reliability total			0.736								

Legend: S: Saturation, C: Communalities, R: Reliability (Cronbach), PV: Proper values, %V: % of the variance.

**Table 4:** Percentiles to classify ABSOW-I (EACAM-I) based on age, BMI and NC.

Indicators	L	M	S	P3	P5	P10	P15	P25	P50	P75	P85	P90	P95	P97
Age (years)														
60.0 to 64.9	−2.83	160.20	0.07	144	146	149	151	154	160	168	173	176	182	186
65.0 to 69.9	−0.40	159.02	0.06	142	144	147	149	152	159	166	170	173	177	180
70.0 to 74.9	1.30	158.81	0.06	140	142	146	148	152	159	166	169	171	175	177
75.0 to 79.9	3.15	153.94	0.06	133	136	141	143	147	154	160	163	165	168	170
>80.0	4.21	136.68	0.06	117	120	125	127	131	137	142	145	146	149	150
BMI (kg/m^2^)														
<26.4	2.77	161.32	0.07	137	141	146	149	154	161	169	172	175	178	180
26.5 to 32.2	1.97	155.88	0.08	132	135	140	143	148	156	164	168	170	174	177
32.3 to 38.0	2.98	155.12	0.07	131	135	140	143	147	155	162	166	168	171	173
>38.1	6.87	153.47	0.06	127	133	139	143	147	154	159	161	163	165	166
C. Neck														
<31.4	1.81	164.79	0.07	142	145	149	152	157	165	172	176	179	183	186
31.5 to 35.8	2.22	157.72	0.07	134	137	142	145	150	158	165	169	172	176	178
35.9 to 39.8	3.34	155.50	0.07	131	135	140	143	148	156	162	166	168	171	173
>39.9	5.36	152.60	0.06	126	131	137	141	145	153	159	162	163	166	167

Legend: BMI: Body Mass Index, C: Circumference, L: Box-Cox Power, M: mean, S: Coefficient of variation.

## Data Availability

The data is available upon request from the authors.

## Supplementary Materials

The following are available online at https://www.mdpi.com/1660-4601/18/2/731/s1, Self-Perceived Adaptive Behaviors Scale
