# Supplementary material for: Validation of a Self-Perceived Adaptive Behaviors Scale in Older Chilean Women and Percentiles for Evaluation
Source: Int J Environ Res Public Health. 2021 Jan 16;18(2):731. doi: 10.3390/ijerph18020731 (PMC7830630; doi:10.3390/ijerph18020731)
Supplement: Supplementary file 1 [file ijerph-18-00731-s001.pdf]

Self-Perceived Adaptive Behaviors Scale

Age:
Gender:
Province:
Nationality:
Instructions:
Mark with an X the answer.
You remember this answer is anonymous.

Self-perception scale for assessing the adaptive behavior of older Chilean women				
	**Comunication**			
1	Do you talk with your family members and friends about your favorite activities?	Always	Sometimes	Never
2	Do you often pay attention to conversations you have in groups and/or with family?	Always	Sometimes	Never
3	Do you often show positive attitudes to motivate your peers?	Always	Sometimes	Never
4	Do you use current topics in your conversations?	Always	Sometimes	Never
5	Do you talk with friends or family about issues related to your future?	Always	Sometimes	Never
	**Use of community resources**			
1	Do you worry about having an up-to-fate telephone list of emergency services (police, fire, and ambulance)?	Always	Sometimes	Never
2	Do you use the computer to write letters to send messages by e-mail to friends and family?	Always	Sometimes	Never
3	Do you know how to give information to someone in order to reach your destination?	Always	Sometimes	Never
4	When you are in a building, do you worry about looking for the exit signs in case of an emergency?	Always	Sometimes	Never
5	Do you use city transportation systems without help?	Always	Sometimes	Never
6	In the city where you live, do you know how to get to a hospital or police station without help?	Always	Sometimes	Never
	**Home life**			
1	Do you use with ease and without help all of the appliances in your home?	Always	Sometimes	Never
2	Do you prepare and cook your food for breakfast, lunch, and dinner?	Always	Sometimes	Never
3	Do you usually clean and wash your clothes, footwear, dishes, among other things?	Always	Sometimes	Never
4	Do you usually clean and wash the bathroom, bedroom, living room, and kitchen in your home?	Always	Sometimes	Never
5	Are you able to wash, iron, and put away your clothes?	Always	Sometimes	Never
	**Health**			
1	If you feel ill, can you get to a clinic or hospital without help?	Always	Sometimes	Never
2	Do you often go for medical checkups?	Always	Sometimes	Never
3	In the past weeks, have you smoked?	Always	Sometimes	Never
4	From your perspective, do you consider your weight to be	Under weight	Normal	Over weighto
5	Do you go to the optometrist and dentist for regular checkups?	Always	Sometimes	Never
	**Safety**			

1	In general, do you know about safety standards, for example, non-smoking areas, flammables, among others?	Always	Sometimes	Never
2	Do you arrange cables or plugs in order to prevent a short circuit?	Always	Sometimes	Never
3	When children are in the house, do you allow them to use the electric appliances?	Always	Sometimes	Never
4	De you have a medicine cabinet in the house in case of a small accident?	Always	Sometimes	Never
5	Do you have a backpack prepared containing utensils, non0perishable food, water (among others) in case of an emergency?	Always	Sometimes	Never
	**Self-care**			
1	Do you usually use hygiene services without help?	Always	Sometimes	Never
2	Do you usually organize your utensils, objects, documents, among others, for activities for the following day?	Always	Sometimes	Never
3	Do you usually use dental floss for dental hygiene?	Always	Sometimes	Never
4	Do you chew your food more than 20 times before swallowing your food??	Always	Sometimes	Never
5	Are you able to cut your nails and clean them?	Always	Sometimes	Never
6	Are you in control of your body weight?	Always	Sometimes	Never
7	Do you usually iron your clothes in order to look presentable?	Always	Sometimes	Never
	**Functional abilities**			
1	Do you read and obey signs, such as NO crossing, exit, entrance, etc.?	Always	Sometimes	Never
2	Do you usually write down important dates, such as birthdays, anniversaries, among others?	Always	Sometimes	Never
3	Do you usually finish reading texts you begin, such as newspapers and books?	Always	Sometimes	Never
4	Do you write letters or messages to send via e-mail?	Always	Sometimes	Never
5	In general, do you know how to fill out forms (such as, for example, civil registry, taxes, among others?	Always	Sometimes	Never
6	Usually, are you interested in reading detailed contracts, letters, forms, among others?	Always	Sometimes	Never
7	Do you know how to make appointments (hourly) for medical consultations?	Always	Sometimes	Never
	**Leisure**			
1	Regularly, do you listen to music in order to relax?	Always	Sometimes	Never
2	In general, do you buy books or magazines to read?	Always	Sometimes	Never
3	Do you usually go to the movies or theatre with friends or family?	Always	Sometimes	Never
4	Are you accustomed to going out to walk in the park or plaza alone or with friends?	Always	Sometimes	Never
5	Do you actively participate in a group, club, church, or others, with friends?	Always	Sometimes	Never
6	Do you go for walks in the country with friends, family, or children?	Always	Sometimes	Never
	**Self-direction**			
1	Do you save money for day-to-day expenses and for the future?	Always	Sometimes	Never
2	At home, do you think positively about taking care of your house (cleaning, washing, arranging, among others)?	Always	Sometimes	Never
3	When you have appointments or meetings, do you usually arrive on time?	Always	Sometimes	Never
4	Do you plan your activities in advance?	Always	Sometimes	Never
5	When you experience setbacks, do you phone home to let people know that you will be delayed or that you will be late?	Always	Sometimes	Never

6	Frequently, do you usually complete activities on time that you schedule?	Always	Sometimes	Never
	**Socialization**			
1	Do you offer to help family or friends?	Always	Sometimes	Never
2	Day to day, do you maintain a stable group of friends that get together?	Always	Sometimes	Never
3	Are you cautious in looking for or accepting friendships?	Always	Sometimes	Never
4	Do you apologize when you say something hurtful to friends or family?	Always	Sometimes	Never
5	Frequently, do you give gifts to family or friends?	Always	Sometimes	Never
	**Functional capacity**			
1	During the week, do you usually walk long distances?	Always	Sometimes	Never
2	Do you use stairs to go up and down?	Always	Sometimes	Never
3	In general, can you pull a suitcase without showing signs of fatigue?	Always	Sometimes	Never
4	When you go to the city center, can you get around easily on a bus or a collective??	Always	Sometimes	Never
5	Do you actually participate actively in a any physical fitness group?	Always	Sometimes	Never

Edad:
Género:
Provincia:
Nationalidad:
Instruccciones: Marque con una X la respuesta.
Recuerda que tu respuesta es anónima.

Self-perception scale for assessing the adaptive behavior of older Chilean women - Spanish				
	**Comunicación**			
1	Hablas con tus familiares y amigos sobre tus actividades preferidas?	Siempre	A veces	Nunca
2	A menudo prestas atención a las conversaciones que tienes en grupo y/o familia?	Siempre	A veces	Nunca
3	Muestra a menudo actitudes positivas para animar a tus semejantes?	Siempre	A veces	Nunca
4	Utilizas temas actuales para tus conversaciones?	Siempre	A veces	Nunca
5	Hablas con tus amigo o familiares sobre cuestiones relacionadas a tu futuro?	Siempre	A veces	Nunca
	**Uso de recursos de la comunidad**			
1	Te preocupas por tener actualizada tu lista de números telefónicos de emergencia (carabineros, bomberos, Ambulancia)?	Siempre	A veces	Nunca
2	Redactas cartas en el compitador para enviar mensaje por e-mail a tus amigos y familiares?	Siempre	A veces	Nunca
3	Sabes proporcionar información a alguien para que peuda llegar a su destino?	Siempre	A veces	Nunca
4	Cuando estás en un edificio, te preocupas por buscar las señalizaciones en caso de que ocurra una emergencia?.	Siempre	A veces	Nunca
5	Usas los medios de transporte de la ciudad sin ayuda?	Siempre	A veces	Nunca
6	En la ciudad donde vives, sabes como llegar a aun hospital, carabineros, sin ayuda?	Siempre	A veces	Nunca
	**Vida en el hogar**			
1	Utilizas con facilidad y sin ayuda todos los electrodomésticos de tu casa?	Siempre	A veces	Nunca
2	Preparas y cocinas tus alimentos para desayunar, almorza y cenar?	Siempre	A veces	Nunca
3	Sueles limpiar y asear tu ropa, calzados, vajilla, entre otros?	Siempre	A veces	Nunca
4	Sueles limpiar,a sear el baño, cuarto, sala y cocina de tu casa?	Siempre	A veces	Nunca
5	Eres capáz de lavar, planchar y ordenar tu ropa?	Siempre	A veces	Nunca
	**Salud**			
1	Si te sientes mal, acudes a un centro médico u hospital sin ayuda?	Siempre	A veces	Nunca
2	Vas a menudo a los controles médicos?	Siempre	A veces	Nunca
3	En las ultimas semanas has fumado?	Siempre	A veces	Nunca
4	Según tu percpeción, consideras que tu peso actual es:	Bajo peso	Normal	Sobrepeso
5	Acudes al oculista y dentistas para tus controles?	Siempre	A veces	Nunca
	**Seguridad**			
1	En general, tienes conocimiento sobre las normas de seguridad, por ejemplo, lugares donde no fumar, inflamables, entre otros?	Siempre	A veces	Nunca
2	Ordenas los cables, enchufes para no generar corto-circuito?	Siempre	A veces	Nunca

3	Cuando hay niños en casa, no dejo que manipulen los electromésticos.	Siempre	A veces	Nunca
4	Tienes un botiquín en casa, en caso que se presente un pequeño accidente?	Siempre	A veces	Nunca
5	Tienes preparado una mochila con utensilios, alimentos no peresibles, agua, entre otros) en caso de un terremoto?	Siempre	A veces	Nunca
	**Autocuidado**			
1	Sueles utilizar los servicios higieneicos sin ayuda?	Siempre	A veces	Nunca
2	A menudo organzas tus utensilios, objetos, documentos, entre otros para las actividades del día siguiente?.	Siempre	A veces	Nunca
3	Utilizas a menudo hilo dental para la higiene de tus dientes?	Siempre	A veces	Nunca
4	Masticas más de 20 veces antes de tragar el bolo alimenticio?	Siempre	A veces	Nunca
5	Eres capaz de cortar las uñas y asearse?	Siempre	A veces	Nunca
6	Llevas el control de peso corporal?	Siempre	A veces	Nunca
7	Sueles planchar tu ropa para adecuada presentación?	Siempre	A veces	Nunca
	**Habilidades funcionales**			
1	Lee y obedece señales, como No pasar, salir, entreda, etc.	Siempre	A veces	Nunca
2	Sueles anotar las fechas importantes, como cumpleaños, aniversarios, entre otros?	Siempre	A veces	Nunca
3	Sueles terminar las lecturas que inicias en cuanto a periódicos y libros?	Siempre	A veces	Nunca
4	Escribes cartas, mensajes para enviar por emdio de correo electrónico?	Siempre	A veces	Nunca
5	En general,s abes llenar formularios (como porejemplo del registro civil, impuestos, entre otros)?	Siempre	A veces	Nunca
6	A menudo te inetesas por ller detenidamente contrattos, cartas, formularios, entre otros?	Siempre	A veces	Nunca
7	Sabes sacar cita (hora) para las consultas médicas?	Siempre	A veces	Nunca
	**Ocio**			
1	Con frecuencia sueles escuchar música para relajarse?	Siempre	A veces	Nunca
2	Por lo general, compras libros, revistas para hacer lectura?	Siempre	A veces	Nunca
3	Sueles asistir al cine, teatro con amigos o familia?	Siempre	A veces	Nunca
4	Acostumbras salir a caminar al parque, plaza sólo o con amigos?	Siempre	A veces	Nunca
5	Participas activamente en un grupo de amigos, club, iglesia, entre otros?	Siempre	A veces	Nunca
6	Realzias paseos campestres con amigos, familaires e hijos?	Siempre	A veces	Nunca
	**Audirección**			
1	Guardas dinero para gastos del día a día y para el futuro?.	Siempre	A veces	Nunca
2	En tu casa muestras actitudes positivas para organziar tu casa (limpieza, aseo, orden, entre otros).?	Siempre	A veces	Nunca
3	Cuando tienes citas o reuniones, sules llegar a la hora?	Siempre	A veces	Nunca
4	Planificas tus actividades con antelación?	Siempre	A veces	Nunca
5	Cuando hay contratiempos, llamas a tu casa para avisar que demorarás o avisas que llegarás tarde?	Siempre	A veces	Nunca
6	Con frecuencia sueles terminar a tiempo las actividades que programas?	Siempre	A veces	Nunca
	**Socialización**			
1	Ofreces ayuda a tus familiares o amigos?	Siempre	A veces	Nunca
2	En el día día, mantienes un grupo estable de amigos que se frecuentan?	Siempre	A veces	Nunca

3	Eres cautelozo para buscar o aceptar tus amistades?.	Siempre	A veces	Nunca
4	Te disculpas en caso de que hayas dicho algo hiriente a tus amigos o familiares?	Siempre	A veces	Nunca
5	Con frecuencia realzias regalos a tus amigos o familiares?	Siempre	A veces	Nunca
	**Capacidad funcional**			
1	A menudo durante los dias de la semana sueles caminar largas distancias?	Siempre	A veces	Nunca
2	Utilizas escaleras para subir y bajar?	Siempre	A veces	Nunca
3	Por lo geenral, puedes jalar una maleta sin mostrar signos de cansancio?.	Siempre	A veces	Nunca
4	Cuando sales al centro de la ciudad, te trasnportas con facilidad en micro o colectivo?	Siempre	A veces	Nunca
5	Actualmente participas activamente del algún grupo que haga actividad física?	Siempre	A veces	Nunca
